# The epigenetic landscape: An evolving concept

**DOI:** 10.3389/freae.2023.1176449

**Published:** 2023-03-20

**Authors:** Steven Henikoff

**Affiliations:** ^1^ Fred Hutchinson Cancer Center, Seattle, WA, United States; ^2^ Howard Hughes Medical Institute, Seattle, WA, United States

**Keywords:** epigenetics, Waddington, epigenomic tools, chromatin, plants, metabolism, cancer

## Abstract

The epigenetic landscape was a visual metaphor introduced in the mid-twentieth century to illustrate the genetic control of embryonic differentiation. Although the popular understanding of epigenetics has since expanded to include gene and chromosomal mechanisms in all contexts, the landscape metaphor provides a unifying concept centered around processes that establish and maintain cellular memory. However, over the decades the term epigenetics has been also used to describe some non-genetic processes that bear little or no resemblance to the traditional concept of an epigenetic landscape. By establishing *Frontiers in Epigenetics and Epigenomics*, we aim to provide authors and readers a forum and an outlet for research that is centered around the original concept of an epigenetic landscape. Thanks in large part to exciting advances in epigenomic technologies, we expect that a deeper understanding of cellular memory will translate into new strategies for medicine, agriculture, and environmental health.

## Main text

“Epigenetics” is the term used by Conrad Hal Waddington in 1942 to describe causal genetic mechanisms that underlie embryonic development (Waddington, 2012), recalling the ancient theory of “epigenesis” in opposition to the preformationist view. Waddington famously illustrated the concept as a marble rolling down a rugged hillside, where valleys of this “epigenetic landscape” were held in place by guy wires underneath attached to pegs in the ground representing genes (Waddington, 1957). Some of Waddington’s own work provided examples of disruptions of the epigenetic landscape, such as briefly heating *Drosophila* pupae to induce a specific defect in the adult wing (Waddington, 1952). Classical examples of epigenetic disruptions included clonal inheritance observed in *Drosophila* position-effect variegation (PEV) (Muller, 1930) and the orange and black patches seen in the fur of calico cats (Kalantry, 2011). Epigenetic disruptions also included violations of stable Mendelian inheritance in maize termed “paramutation,” where an allele is heritably transformed in a diploid heterozygote to yield progeny resembling those of the alternative allele (Hollick et al., 1997). These examples of disruptions of the chromatin landscape and others like them observed in diverse eukaryotes have inspired many researchers to explore their underlying molecular mechanisms. From this historical perspective, the field of epigenetics is the study of cellular memory detected phenotypically by heritable mitotic or meiotic disruptions.

These classical examples of epigenetic disruptions predated the birth of molecular biology, which ushered in a rather different meaning of epigenetics from Waddington’s. As DNA is carrier of genetic information, and epigenetics literally means “above” genetics, epigenetic information would be that maintained by non-DNA components of chromosomes (Haig, 2004). Accordingly, the term epigenetics has been used to describe the action of proteins that mediate and regulate gene expression. Rapid progress in understanding the nuts-and-bolts of gene expression in the 1950s and 1960s led to the view of epigenetics as synonymous with the study of the chromosomal proteins and complexes involved in gene regulation (Ptashne, 2004). In this way, epigenetic phenomena included the binding of general and sequence-specific transcription factors guiding the initiation of transcription by RNA polymerases. The role of transcription factors in controlling the epigenetic landscape and mediating cellular memory was later confirmed by demonstrations that master regulatory transcription factors such as MyoD (Tapscott et al., 1988) and Yamanaka factors (Takahashi and Yamanaka, 2006) could initiate switches in cell fate.

However, the epigenetic landscape encompasses orders of magnitude more of the chromosome than gene transcription units, only a small part of which have evolved for gene regulation during development. Transposable elements and their remnants occupy nearly half of our DNA, and vast non-genic stretches are found around centromeres and telomeres; preventing unscheduled transcription in these regions is critical to maintain integrity of the epigenetic landscape. Silencing of these elements in many animals and plants is mediated by DNA methylation, maintained epigenetically by the DNMT1 DNA methyltransferase, which efficiently methylates hemi-methylated CG dinucleotides behind the replication fork (Holliday, 1996). The discovery of nucleosomes in the early 1970s, and their regional differentiation by histone modifications and variants, led to the realization that nucleosomes and the machines that deposit, modify and remodel them are central players in epigenetic regulation (Jenuwein and Allis, 2001). For example, the classical epigenetic phenomenon of PEV is now understood to be “spreading” of chromatin-associated proteins mediated by methylation of lysine-9 on histone H3 and by binding of HP1 and other heterochromatin-associated proteins (Grewal and Jia, 2007). As we learn more about chromatin regulation, we have come to better appreciate the importance of co-activators and co-repressors that act through nucleosomes to regulate the epigenetic landscape. Thus, over the decades since Waddington first introduced the epigenetic landscape, epigenetic studies and “epigenomic” technologies have come to represent a coherent field of genetics research that is central to understanding cell biology and development.

My interest in epigenetics began as a post-doc studying *Drosophila* PEV, which later led to my participation in the first Epigenetics Gordon Conference in 1995. Organized by Vicki Chandler, who studied paramutation in plants (Hollick et al., 1997) and Tim Bestor, who studied mammalian DNA methylation (Bestor, 1996), the first order of business for this diverse gathering was to clarify what epigenetics is. The variety of perspectives at the time reflected in part confusion over the epigenesis and “above-genetics” derivations of the term but mostly the difficulty in identifying common molecular mechanisms that unite the various animal, plant and fungal phenomena that fall under the epigenetics umbrella. Fortunately, the application of emerging genome-wide and imaging technologies has since provided mechanistic explanations for these seemingly mysterious phenomena. But as we learn more about epigenetic mechanisms, new questions and challenges arise. Perhaps no better example is the realization that cancer is not only a genetic disease, driven by mutations in oncogenes and tumor suppressor genes, but also has an epigenetic component, in that driver mutations often result in profound global changes in the epigenetic landscape. Indeed cancer epigenetics, the subject of speculation less than 20 years ago (Feinberg et al., 2006) has become one of the fastest growing fields of epigenetic research (Armstrong et al., 2017). This impression is supported by the text-mining map of topics in epigenetics research displayed in Figure 1, where almost the entire right half of the graph is associated with cancer and cancer treatments.

**FIGURE 1:**
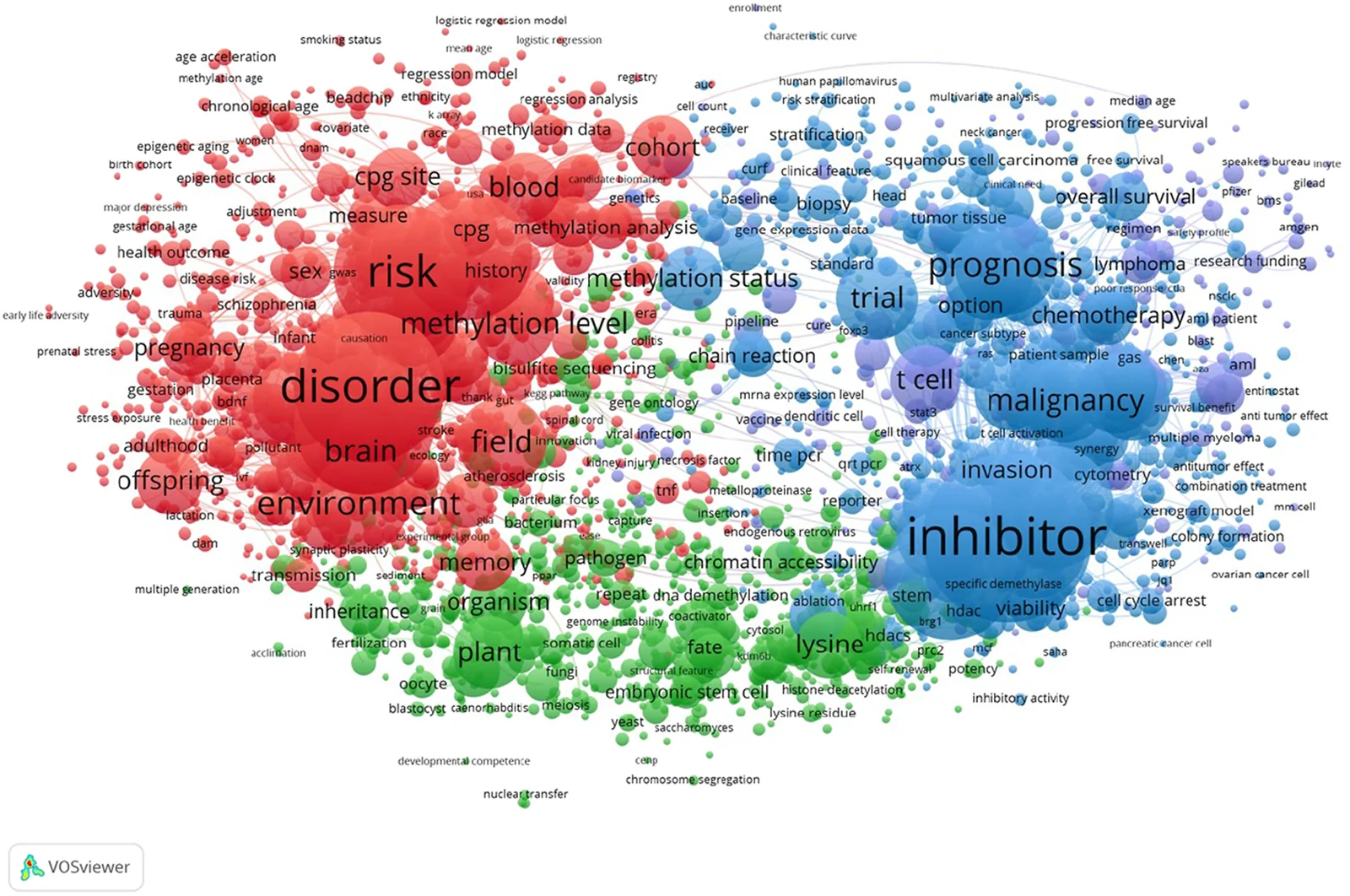
A bibliometric map of epigenetics-based publications. Vosviewer (van Eck and Waltman, 2010) was used to generate an association map, where nodes were clustered based on similarity, with node area proportional to the number of documents (*N* = 56,958) retrieved using titles and abstracts of 2017–2021 publications. Keywords used were: Epigenetics, Histone modifications, Cancer Epigenetics, Epigenetics Inhibitors, Epigenetics Therapeutics, Inherited Epigenetics, Environmental Epigenetics, Epigenomics, Cancer Epigenomics, Epigenetic Therapy, Epigenomic Therapy. Strongly related topics are highlighted in color, where red includes inherited and environmental Epigenetics, blue includes cancer epigenetics, inhibitors and therapeutics.

The text-mining map also illustrates how far afield epigenetics has veered from its Waddingtonian origins. The term “environmental versus inherited epigenetics” is especially diffuse and accounts for most of the left half of the graph. A major component of this topic is “risk”, despite the relative dearth of evidence that non-genetic disease risk results from dysfunction of cellular memory processes. This increasing heterogeneity of what is thought to be epigenetic presents a challenge for editors of journals devoted to the topic. For example, when in 2008 Frank Grosveld and I founded the Biomed Central journal, *Epigenetics and Chromatin*, we noted a 15-fold increase over the previous decade in articles given the Pubmed subject heading “epigenetics” (Henikoff and Grosveld, 2008). Our goal then was to provide an open access outlet for studies exploring the intersection of chromatin, gene regulation and cellular memory. In the 15 years since, the proliferation of studies considered by their authors to be epigenetic continues, and as a result, one hardly knows nowadays what is and what is not considered to be epigenetic. A grand challenge for the Editors of *Frontiers in Epigenetics and Epigenomics* is to attract submissions that fall within the epigenetic landscape paradigm for the benefit of both authors and readers.

Another grand challenge that the Editors of *Frontiers in Epigenetics and Epigenomics* face are changes in the journal publication industry driven by the growing popularity of preprint servers such as *bioRxiv* (https://www.biorxiv.org) which did not exist when Frank Grosveld and I began *Epigenetics and Chromatin*. An author’s ability to make a manuscript public on the internet at one’s own discretion has changed the way many of us view the publication process. A manuscript posted on *bioRxiv* gets immediate attention, and the scientific enterprise moves forward without the delays imposed by the need for peer review and editorial decisions at each step. Recently the PubMed catalog has started to include *bioRxiv* manuscripts supported by grants from the National Institutes of Health, so that the practical distinction between a peer-reviewed publication and a preprint has diminished. Also, it is now common for funding organizations and faculty promotion committees to accept preprints as evidence of productivity, further encouraging the posting of preprints. Journals must therefore provide sufficient value in the form of peer review and prestige to justify their open access fees.

One solution to the challenge posed by preprint servers is for a publisher to post a submission immediately on a preprint server and post reviews in the comment box, thus relieving authors of uncertainty while rewarding reviewers by publishing their reviews regardless of whether or not the manuscript is accepted by that journal. I first heard the idea of using the *bioRxiv* comment box for one’s own review of the manuscript from Sophien Kamoun at dinner following a Scientific Advisory Board meeting in 2017 (https://zenodo.org/record/1466784#.Y-llzC-B3BB). Posting of a review could be most readily achieved by the journal itself, thus providing provenance for the review while maintaining anonymity. Implementation of Kamoun’s idea was not feasible for *Epigenetics and Chromatin*, which is published by Springer-Nature, however *eLife* has recently adopted a policy of posting reviews with an editorial assessment for all submissions sent out for review (Eisen et al., 2022).

While preprints accompanied by reviews in the *eLife* model and other post-publication peer review models provide rewards for authors, and reviewers can get name recognition for their work in the supplementary decision letter, it is less clear how important this additional document is for readers. Biomedical scientists are already accustomed to look out for preprints in their areas of interest, and *bioRxiv* allows updated versions prior to journal acceptance, so that by the time the peer review document appears online, it is unclear as to how many readers would bother to look at it. Some peer review reports, rebuttals and editorial assessments might be of interest to future historians, but it is hard to envision just how this documentation will be of value to researchers once the article is finalized and the *bioRxiv* preprint is linked to the final publication.

*Frontiers* journals use an online publication model that further emphasizes recognition for the peer reviewers and the editors who collaborated with the authors during the peer review process. First, by ascertaining only quality and not perceived impact, reviewers and editors are spared from making subjective judgments that might make them uneasy about revealing their identities, especially if they feel professionally vulnerable. Second, a paper rejected for insufficient quality is not saddled with the permanent stigma of negative post-publication reviews. Third and most importantly, reviewers and editors are prominently identified on the first page of the article, including their photos and links to their publications, reviewing activities and biographical information, rewarding them upfront for their efforts. By thus bringing together authors, reviewers and editors of every published manuscript, *Frontiers in Epigenetics and Epigenomics* aims to become a true community journal.

The journal’s four interrelated sections span the breadth of this rapidly expanding field: Chromatin Epigenetics encompasses fundamental processes and mechanisms whereby complexes of DNA, proteins and RNA and their modifications mediate cellular memory. Epigenetics and Metabolism includes a broad range of topics such as signals from the external and intracellular environment that modify the epigenome in health and disease. Plant Epigenetics describes the diverse plant-specific cell memory phenomena that have broadened and deepened our understanding of fundamental gene and chromosome regulatory processes. Epigenomic Tools include genomic, imaging and other technologies that continue to drive chromatin and epigenetic research, a broad topic that thus far has been under-represented in related *Frontiers* journals. Together with the Section Chief Editors and their networks of Associate Editors, we aim to make the experience of publishing in and reviewing for *Frontiers in Epigenetics* and *Epigenomics* a positive experience.

## Data Availability

The original contributions presented in the study are included in the article/Supplementary Material, further inquiries can be directed to the corresponding author.
